# Associating Automated Breast Ultrasound (ABUS) and Digital Breast Tomosynthesis (DBT) with Full-Field Digital Mammography (FFDM) in Clinical Practice in Cases of Women with Dense Breast Tissue

**DOI:** 10.3390/diagnostics12020459

**Published:** 2022-02-11

**Authors:** Ioana Boca (Bene), Anca Ileana Ciurea, Ștefan Cristian Vesa, Cristiana Augusta Ciortea, Sorin Marian Dudea, Simona Manole

**Affiliations:** 1 Department of Radiology, “Iuliu Hatieganu” University of Medicine and Pharmacy, 400012 Cluj-Napoca, Romania; ioanaboca90@yahoo.com (I.B.); sdudea1@gmail.com (S.M.D.); simona.manole@gmail.com (S.M.); 2 Department of Pharmacology, Toxicology and Clinical Pharmacology, “Iuliu Hatieganu” University of Medicine and Pharmacy, 400012 Cluj-Napoca, Romania; stefanvesa@gmail.com; 3 Department of Radiology, Emergency County Hospital, 400006 Cluj-Napoca, Romania; cristianaciortea@yahoo.com

**Keywords:** full-field digital mammography (FFDM), digital breast tomosynthesis (DBT), automated breast ultrasound (ABUS), dense breast tissue

## Abstract

The purpose of the present study was to evaluate the value of full-field digital mammography (FFDM) and automated breast ultrasound (ABUS) in the diagnosis of breast cancer compared to FFDM associated with digital breast tomosynthesis (DBT). Methods: This retrospective study included 50 female patients with a denser framework of connective tissue fibers, characteristic of young women who underwent FFDM, DBT, handheld ultrasound (HHUS), and ABUS between January 2017 and October 2018. The sensitivity (Se), specificity (Sp), positive predictive value (PPV), negative predictive value (NPV), and accuracy of FFDM+ABUS were 81.82% (95% CI [48.22–97.72]), 89.74% (95% CI [75.78–97.13]), 69.23% (95% CI [46.05–85.57]), 94.59% (95% CI [83.26–98.40]), and 88% (95% CI [75.69–95.47]), while for FFDM+DBT, the values were as follows: 91.67% (95% CI [61.52–99.79]), 71.79% (95% CI [55.13–85.00]), 50% (95% CI [37.08–62.92]), 96.55% (95% CI [80.93–99.46]), 76.47% (95% CI [62.51–87.21]). We found an almost perfect agreement between the two readers regarding FFDM associated with ABUS, and substantial agreement regarding FFDM+DBT, with a kappa coefficient of 0.896 and 0.8, respectively; *p* < 0.001. Conclusions: ABUS and DBT are suitable as additional diagnostic imaging techniques to FFDM in women at an intermediate risk of developing breast cancer through the presence of dense breast tissue. In this study, DBT reduced the number of false negative results, while the use of ABUS resulted in an increase in specificity.

## 1. Introduction

Mammography is the basic and most frequently used standard method currently adopted for breast cancer screening [1]. In cases of positive screening mammography or in symptomatic patients, the diagnostic evaluation may consist of additional mammographic views (spot compression, magnification), ultrasound, and even contrast-enhanced imaging techniques such as dynamic contrast-enhanced magnetic resonance imaging (DCE MRI) or contrast-enhanced spectral mammography (CESM).

In the United States, 40% of women have dense breasts, and a third of breast cancers in these cases are mammographically occult, having an increased risk of developing breast cancer compared to women with fatty breasts [2]. Following the implementation of statewide breast density reporting laws, Weigert et al. [3] began a multicenter retrospective study in 12 locations in Connecticut. They included 72,030 screening mammograms and 8647 screening ultrasounds and evaluated the performance of additional ultrasound screening in women with dense breasts and a normal mammographic appearance. The sensitivity was 96.6%, the specificity was 94.9%, and they detected 28 additional cancers (3.25 cancers per 1000 women screened), with a 14% recall rate. The results are similar to those obtained by Berg et al. [4] in the ACRIN 6666 trial, which obtained a sensitivity of 76%, a specificity of 84%, and 3.70 additional cancers detected per 1000 examinations. Although the American Cancer Society (ACS) recommends the use of additional magnetic resonance imaging (MRI) screening in the case of women at a high risk of developing breast cancer, the role of additional screening in the case of women at an intermediate risk and with an increased breast density is still unclear [5]. Over time, numerous studies have been conducted to improve the diagnostic performance.

DBT has begun to be used to overcome the limitations of FFDM. DBT has the ability to overcome tissue superimposition effects and achieve better lesion conspicuity and margins, thus providing advantages in assessing masses, areas of architectural distortion, and asymmetries compared to FFDM; therefore, DBT could reduce the necessity for supplemental views [6].

Automated breast ultrasound (ABUS) represents an ultrasound technique approved by the Food and Drug Administration (FDA) in 2012 for full breast ultrasound screening in cases of women with dense breasts [7]. It has been shown to be effective both as an additional method in screening by increasing the detection rate of breast cancer, and in diagnosis, having a comparable effectiveness to handheld ultrasound (HHUS) [8,9].

Women with dense breasts have an increased risk of developing breast cancer, while mammography has a low sensitivity in these cases. In order to overcome these limitations, using an additional method such as ABUS or digital breast tomosynthesis (DBT) could improve the detection rate of breast cancer. Given that FFDM represents the baseline examination in our institute, the purpose of the present study was to evaluate the value of FFDM and ABUS in the diagnosis of breast cancer, compared to FFDM and DBT.

## 2. Materials and Methods

This retrospective study was approved by the institutional review board (Number/Date: 280/11.08.2020), and informed consent was obtained from each patient before performing DBT and ABUS.

### 2.1. Study Design and Population

Between January 2017 and October 2018, images of 155 female patients who underwent ABUS examinations were retrieved from the database.

The study was conducted on the consecutive ABUS examinations that were acquired only for diagnostic purposes. The indications for performing ABUS examinations were evaluation of a palpable mass, nipple discharge, mastodynia, postsurgical induration or tenderness, and clinical equivocal findings after surgery for breast carcinoma.

ABUS examinations were not performed in patients presented for screening, or patients with a known breast cancer presented for re-evaluation during neoadjuvant chemotherapy (NAC).

All patients included in the study had the following techniques performed in the same session: FFDM, DBT, handheld ultrasound (HHUS), and automated breast ultrasound (ABUS). DBT was performed according to the protocol of our department, in which all symptomatic women or women with dense breasts referred for screening are evaluated through DBT. HHUS was performed independently of ABUS in order to have a double control and to make sure that, through ABUS, we did not lose peripheral or small breast lesions.

Breast density was assessed by two radiologists on the low-energy images using the American College of Radiology (ACR) Breast Imaging-Reporting and Data System (BI-RADS) and classified as follows: A—almost entirely fatty; B—scattered areas of fibroglandular densities; C—heterogeneously dense; D—extremely dense.

**Figure 1:**
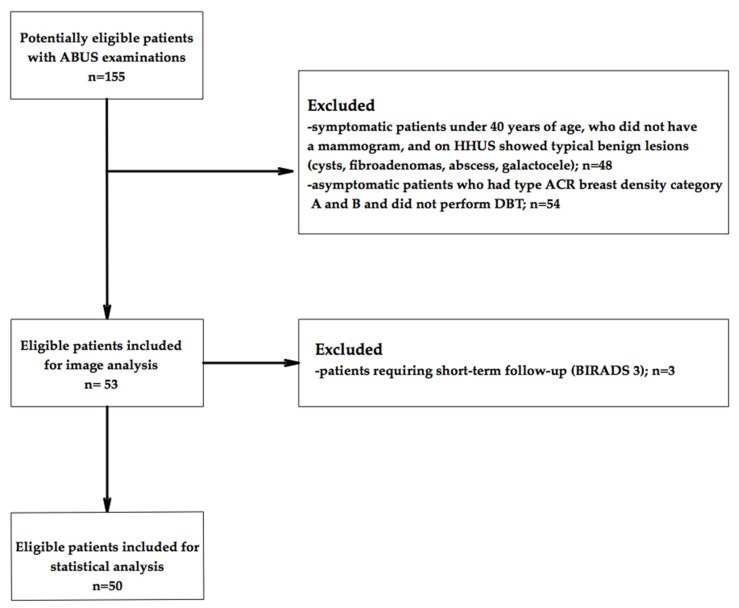
Flowchart of patient inclusion in this study.

Figure 1 summarizes the flowchart of patient selection.

ABUS and mammographic images were reviewed and cross-referenced with the medical data by one radiologist, who did not subsequently participate in the image analysis and statistical analysis of the database.

### 2.2. Image Acquisition

The mammographic examinations were carried out using a Senographe Essential™ mammography unit (General Electric, Buc, France). For each breast, FFDM and DBT were acquired in the mediolateral oblique (MLO) and craniocaudal (CC) views.

HHUS scans were performed by two physicians with extensive experience in breast imaging, using a LOGIQ S8 machine (General Electric, Buc, France).

The automated breast ultrasound was performed with an Invenia ABUS System (General Electric, Buc, France). Images were acquired by a radiographer with appropriate training, so that the examination could be performed correctly, in order to evaluate the entire breast and avoid artifacts. The patients were placed in the dorsal decubitus position with the arm raised above the head. The central, middle, and lateral regions were scanned for each breast (marking the position of the nipple after each scan). Once the entire surface of the breast was scanned in this way, the examination was considered complete. In contrast, additional images were acquired by scanning the upper and lower portions of the breast, if considered necessary.

### 2.3. Image Review

The examinations were independently reviewed by two radiologists with over 20 years of experience in breast imaging. First, the examiners evaluated the FFDMs and the DBT images, establishing a BI-RADS score, taking into account both examinations. In another session, the examiners evaluated the FFDMs and then the ABUS images, establishing another BI-RADS score. The patients with lesions classified as BI-RADS 3 at ABUS were excluded from the study, due to the necessity of short-term follow-up; in these cases, the follow-up was carried out with HHUS.

During the reading sessions, the examiners had no clinical information regarding the patients and no previous images for comparison. In case of inconsistencies, the two parties re-evaluated the images in a separate meeting and agreed on a BI-RADS score.

### 2.4. Reference Standard

We considered as standard: the result of the pathological examination for the biopsied lesions, the typical benign aspect of the lesions (cysts, intramammary lymph node), and the unchanged features at HHUS after 2 years of the solid lesions with typical morphology for a fibroadenoma. All patients were re-evaluated 2 years later, and no further cancer was detected.

### 2.5. Statistical Analysis

Statistical analysis was performed using the MedCalc^®^ Statistical Software version 20.011 (MedCalc Software Ltd., Ostend, Belgium; https://www.medcalc.org). Nominal variables were characterized by frequency and percentage. Cohen’s kappa coefficient was used in order to assess the agreement between two imagistic methods and for inter-rater agreement. A *p* value < 0.05 was considered statistically significant. The sensitivity, specificity, positive predictive value, negative predictive value, and accuracy were calculated for each method.

## 3. Results

### 3.1. Study Population

A total of 50 patients with dense breast tissue (ACR breast density categories C and D) were included in the study. Participants’ ages ranged from 46.5 to 60.9 years (median = 50.75). Eleven patients (22%) had malignant breast lesions (invasive ductal carcinomas, no special type; five luminal A subtypes, and six luminal B subtypes) confirmed by ultrasound-guided biopsy.

**Figure 2:**
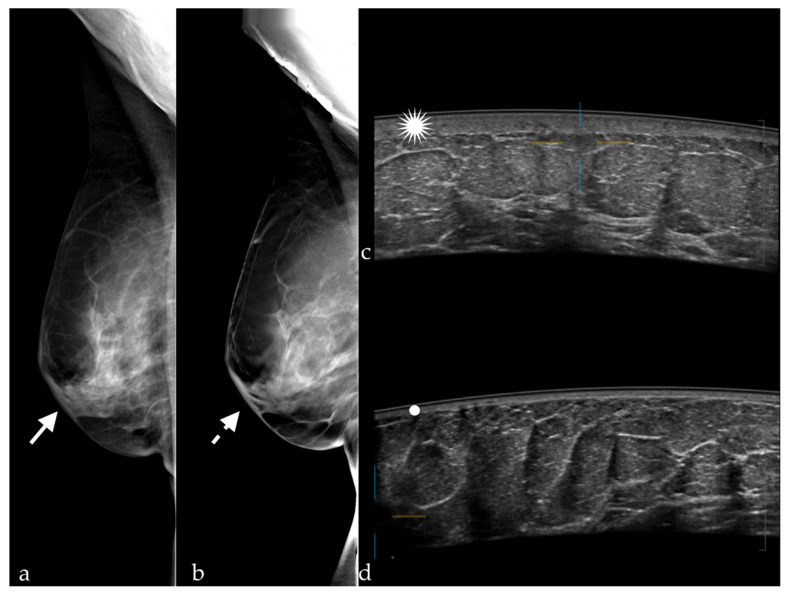
Skin thickening visible on (**a**) FFDM (white arrow) and (**b**) DBT (white dotted arrow) in MLO views, and at (**c**) ABUS (white star); (**d**) normal skin thickness of the left breast (white dot).

The number of BI-RADS 0 cases varied depending on the method used (Table 1). After the interpretation of FFDMs and DBT images, and after the interpretation of FFDMs and ABUS images, only one patient had an indication for further investigations. The findings in this case were non-specific skin thickening and subcutaneous edema due to previous radiotherapy (Figure 2).

Table 2 shows the diagnostic performance of FFDM+DBT and FFDM+ABUS, in terms of sensitivity (Se), specificity (Sp), positive predictive value (PPV), negative predictive value (NPV), and accuracy.

### 3.2. Performance of FFDM+DBT and FFDM+ABUS Compared with the Standard

DBT had moderate agreement with the standard (see Table 3), and there were no false negative results.

Table 4 shows substantial agreement between FFDM+ABUS and the standard. In this group, nine cancers were detected correctly.

### 3.3. Agreement of FFDM+DBT with FFDM+ABUS Interpretation

The absolute numbers of agreement and disagreement for FFDM+DBT and FFDM+ABUS are shown in Table 5, presenting substantial agreement regarding these approaches.

### 3.4. Interobserver Agreement of FFDM+ABUS and FFDM+DBT Interpretation

We found an almost perfect agreement between the two readers regarding FFDM associated with ABUS, and substantial agreement regarding FFDM+DBT, with a kappa coefficient of 0.896 and 0.8, respectively; *p* < 0.001 (see Table 6 and Table 7).

## 4. Discussion

Mammography is the gold standard technique used in the detection of breast cancer. DBT can significantly improve the diagnostic accuracy for noncalcified lesions compared to additional mammographic views [10]. Friedewald et al. [11] conducted a single-reading retrospective cohort study involving 13 breast centers from the United States, including 454,850 examinations (173,663 FFDM+DBT and 281,187 FFDM-only), and reported that adding DBT increased the rate of invasive breast cancer detection from 2.9 per 1000 screened patients (with FFDM-alone) to 4.1 per 1000 screened patients (after associating FFDM with DBT). They did not find a significant difference in the rate of ductal carcinoma in situ (DCIS), suggesting that the cancers detected using DBT may be more clinically important. Rafferty et al. [12] published an additional analysis of data from Friedewald et al. [11], reporting that the association of FFDM with DBT increased the rate of breast cancer detection in women with heterogeneously dense breasts or extremely dense breasts, and that the recall rate was higher in women with heterogeneously dense breasts. In our study, the need for additional evaluation was significantly different compared to the literature, with the STORM trial [13] reporting 4.2%, the OSLO trial [2] 6.7%, and Friedewald [11] 10.7% for FFDM+DBT. However, the data maintain the declining trend after adding DBT (3.6, 6.1, and 9.1%, respectively). The explanation in our study could be that the study group is significantly smaller compared to the large trials mentioned, we included only symptomatic women, and the frequency of malignant lesions was highly correlated with the studied sample. Hakim et al. [14] reported that DBT eliminated the use of ultrasound in the diagnostic process in 32% of cases, results that are concordant with ours (the need for an additional ultrasound dropped by 32% after adding DBT to FFDM).

**Figure 3:**
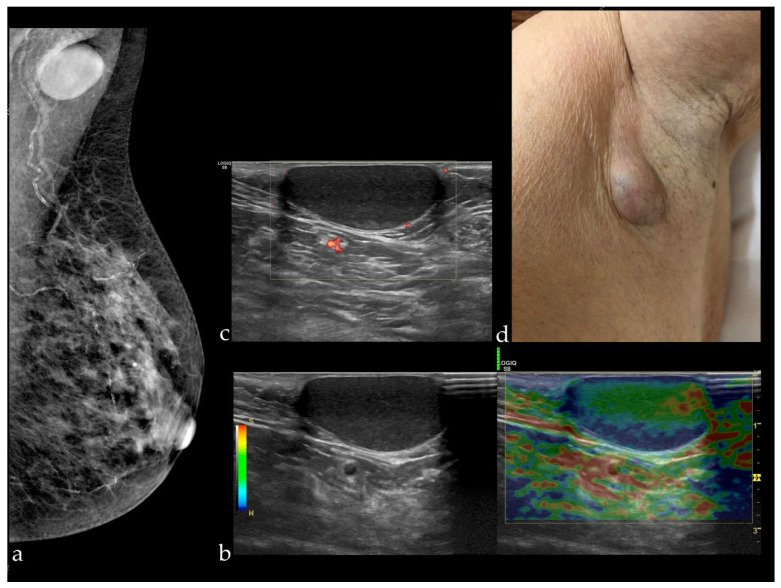
(**a**) Mammography of the left breast in MLO view presenting a circumscribed mass in the left axilla; HHUS revealing a circumscribed cutaneous cystic lesion, soft at elastography (**b**) and non-vascularized (there are visible only some vessels in the surrounding tissue) (**c**), which appeared as a bulging mass at clinical examination (**d**).

Waldherr et al. [15] reported a sensitivity of FFDM+DBT similar to that found in our study (91.9%), but a slightly increased specificity of 87.5%. The STORM trial conducted by Houssami et al. [13], which was a prospective study including 7292 women without symptoms, with an average risk, and aged over 48 years, reported a sensitivity of 85% and a specificity of 97%. Different values were reported in a study performed on 113 patients with 119 breast lesions, following which the average sensitivity and specificity of the three readers involved (with 14, 8, and 8 years of experience) were 97.3 and 44.7%, respectively [16]. In our study, the sensitivity obtained was higher for FFDM+DBT than for FFDM+ABUS (91.67 and 81.82%, respectively). The explanation in this case is given by the fact that all the malignant lesions were associated with architectural distortion, which was detected at DBT. In the case of FFDM+ABUS, a cancer was missed because it appeared in the form of a discrete architectural distortion visible only at DBT, while at ABUS, the lesion was not detected due to the small size of 3 mm. The false positives after FFDM+DBT in our study were represented by eight BI-RADS 0 results, including one case which appeared as architectural distortion on DBT but was just a postsurgical scar, a small fibroadenoma with apparent angulated margins, and an epidermoid cyst in the axilla, mimicking a suspect lymph node (Figure 3).

The use of DBT as an additional examination to FFDM has raised concerns regarding radiation exposure. Wallis et al. [17] reported a similar irradiation dose of FFDM (0.6 mGy/exposure (0.2–1.9 mGy, Sweden, Sectra Mamea system)) and DBT (0.7 mGy/exposure (0.28–1.42 mGy, Sweden), 0.82 mGy/exposure (0.40–1.26 mGy, England)). When DBT was combined with FFDM, the average glandular dose varied, depending on the number of views acquired, from 1.63 mGy for one-view DBT [18] to 3.52 mGy ± 1.08 for two-view DBT+FFDM [19]. The combination of DBT+FFDM leads to twice the radiation dose compared to FFDM, but this increased dose still remains below the FDA limit for a screening examination, which is restricted to 3 mGy per acquisition. In addition, the FDA have approved software for the reconstruction of 2D synthetic views from 3D acquired data, which allows a reduction in the radiation dose to levels comparable to those of FFDM mammography [20]. The interpretation time is significantly higher for DBT-FFDM compared to FFDM alone. A prospective study included 10 radiologists (with 1.5 to 21 years of experience) that read 2163 FFDM and 1502 FFDM-DBT studies in a manner similar to a normal clinical workflow. The average interpretation time was 1.9 min for FFDM and 2.8 min for the combined method, but the study reported a decrease in the interpretation time for FFDM+DBT with increased radiologist experience [21]. Considering the mentioned irradiation aspects, which are acceptable, the fact that this additional examination does not significantly increase the interpretation time and that the NPV obtained in our study is high (96.55%) supports the idea that the use of FFDM+DBT for diagnostic purposes is feasible. Elements suggestive of a previous surgery such as architectural distortion, the presence of surgical clips, or cytosteatonecrosis were easily diagnosed at DBT, while at ABUS, they were interpreted as suspicious, especially in the context of our study, in which the two readers had no clinical data and no information about the patient’s history.

Kim et al. [16] reported that DBT had a similar diagnostic performance to ultrasound regarding characterization of lesions detected on FFDM, but it was not able to distinguish whether a circumscribed mass was a cyst or a complex cystic mass. Ultrasound is able to differentiate between them easily. In addition, many of the cancers detected by ultrasound are small, invasive, and node-negative, but due to the main disadvantages of the method (operator-dependent, time-consuming, cannot be standardized, and high rate of false positive results), studies have been conducted to achieve an efficient workflow by replacing handheld ultrasound (HHUS) with automated breast ultrasound (ABUS) [22,23,24].

In addition, Kim et al. [25] found substantial agreement between ABUS and HHUS (k = 0.773 ± 0.104) regarding the description and characterization of lesions (shape, orientation, edges, delimitation, echo pattern, post-echo features, and calcifications). The most discordant features were those related to posterior echo aspects (k = 0.371 ± 0.225), while the most concordant ones related to orientation (parallel or perpendicular to the skin, k = 0.608 ± 0.201). Golatta et al. [26], in a study that included 913 patients, reported a fair agreement between the two ultrasound methods, k = 0.31 (95% CI [0.26; 0.36]).

The association of ABUS with FFDM significantly improved the detection of breast cancer in women with dense breast tissue without a substantial impairment of specificity (Se = 78.5% and Sp = 76.1%) [5]. The SomoInsight study [8], a prospective multicenter trial that included 15,318 asymptomatic women with dense breasts, compared FFDM with FFDM+ABUS, obtaining a sensitivity of 100% and a specificity of 72% for the association of the two imaging techniques. The cancer detection rate per 1000 screened women increased from 5.4 for FFDM to 7.3 for FFDM+ABUS. Cancers detected with ABUS alone were significantly more likely to be invasive compared to those detected by mammography alone (93.3% versus 62.2%; *p* = 0.001), which has positive prognostic implications.

**Figure 4:**
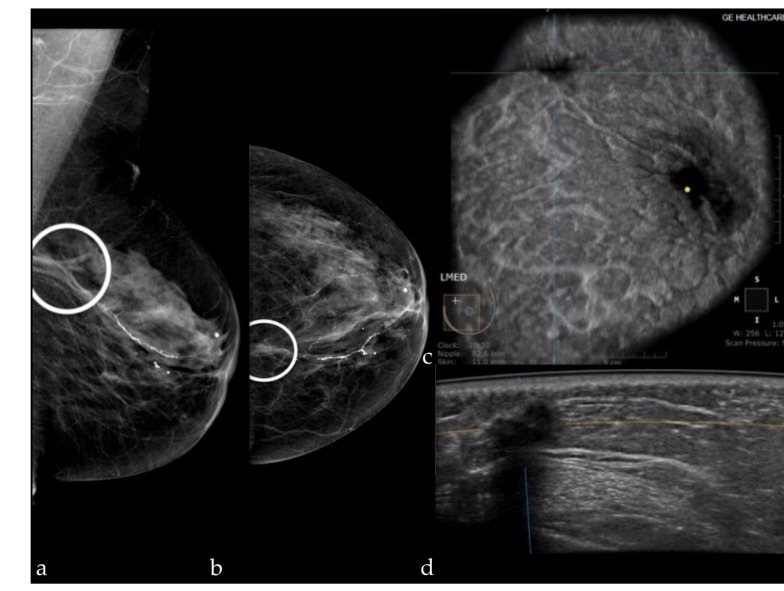
Mammography of the left breast in the MLO view (**a**) and the CC view (**b**) shows an architectural distortion (white circle); ABUS image in coronal plane (**c**) and axial scan (**d**) demonstrating a hypoechoic ill-defined mass with attenuation—surgical scar.

Our results are similar to those described in the literature. Kelly et al. [27] included 4419 women and found a sensitivity of 81% and a specificity of 98.2%. Giuliano et al. [28] included 3418 women (Se = 96.7%; Sp of 98.2%), Wilczek et al. [29] included 1668 women (Se = 100%; Sp = 98.4%), and Giger et al. [30] included 185 women (Se = 74.1%; Sp = 76.2%). The specificity of FFDM+ABUS in our study was higher than that of FFMD+DBT (89.74 and 71.79%, respectively), because the number of false positive results was lower, which is preferable because further work-up is avoided. The false positive results in our study were represented by one BI-RADS 0 score for post radiotherapy skin thickening, in a patient with postsurgical changes (Figure 4). A small fibroadenoma and an epidermal cyst which appeared as a suspect lymph node were detected on the mammography, but due to the location in the axilla, they were not detected at ABUS. These false positive results can be reduced by increasing the experience of the examiners, and by the existence of information related to the clinical history of the patients, although in our study, the examiners did not have access to these data.

The false negative results were represented by two malignant lesions of a small size (5 and 3 mm), which were detected on DBT due to the associated architectural distortion.

The biggest advantage of ABUS is the time saved by the radiologist. While in the case of HHUS, the examiners need 20 min to perform the ultrasound, in the case of ABUS, only the time given to the interpretation is needed. Different interpretation times have been reported in the literature, varying between 2 and 5.2 min depending on the complexity of the cases and the experience of the examiners, but the learning curve for ABUS interpretation is similar to other techniques [26,31].

In our study, we obtained substantial agreement between FFDM+ABUS and the standard, and moderate agreement between FFDM+DBT and the standard, whereas, concerning the interobserver agreement, we found an almost perfect agreement between the two readers regarding FFDM+ABUS, and substantial agreement regarding FFDM+DBT.

Therefore, both methods could be implemented for diagnostic purposes in women with dense breast tissue, each with its advantages and disadvantages. Combining ABUS with FFDM reduces irradiation and the examination time. The shorter time spent by the physician simply reading ABUS images is important, especially in situations where ABUS replaces HHUS, particularly in the services in which HHUS is performed by physicians. In contrast, the association of DBT with FFDM decreases the risk of missing cancers, mainly those that are associated with architectural distortion, even if the architectural distortion is subtle. In addition, the diagnostic performance of both FFDM+DBT and FFDM+ABUS would be increased if physicians had an information sheet related to clinical data and patient history.

We acknowledge that our study has some limitations. It is a single-institution retrospective study. The sample size is small, limiting accurate analysis. The radiologists who reviewed the images had no information regarding the history of the patients or the clinical data, and they had no previous images for comparison, which increased the false positive rate.

## 5. Conclusions

ABUS and DBT are suitable as additional diagnostic imaging techniques to FFDM in women at an intermediate risk of developing breast cancer through the presence of dense breast tissue. In this study, DBT reduced the false negative results, while the use of ABUS resulted in an increase in specificity.

## Figures and Tables

**Table 1:** BI-RADS score results depending on the imaging method used.

BI-RADS Score	Imaging Technique	
	FFDM+DBT	FFDM+ABUS
0	8 (16%)	1 (2%)
1	9 (18%)	14 (28%)
2	19 (38%)	23 (46%)
4	7 (14%)	4 (8%)
5	7 (14%)	8 (16%)

**Table 2:** Diagnostic performance of the imaging techniques.

	Technique	
	FFDM+DBT (95% CI)	FFDM+ABUS (95% CI)
Se (%)	91.67 [61.52–99.79]	81.82 [48.22–97.72]
Sp (%)	71.79 [55.13–85.00]	89.74 [75.78–97.13]
PPV (%)	50 [37.08–62.92]	69.23 [46.05–85.57]
NPV (%)	96.55 [80.93–99.46]	94.59 [83.26–98.40]
Accuracy	76.47 [62.51–87.21]	88 [75.69–95.47]

**Table 3:** FFDM+DBT versus standard.

FFDM+DBT	Standard		Total (%)
	BI-RADS 1 + 2 (%)	BI-RADS 4 + 5 (%)	
BI-RADS 1 + 2 (%)	28 (100)	0 (0)	28 (100)
BI-RADS 0 + 4 + 5 (%)	11 (50)	11 (50)	22 (100)

Kappa 0.554, *p* < 0.0001.

**Table 4:** FFDM+ABUS versus standard.

FFDM+ABUS	Standard		Total (%)
	BI-RADS 1 + 2 (%)	BI-RADS 4 + 5 (%)	
BI-RADS 1 + 2 (%)	35 (94.6)	2 (5.4)	37 (100)
BI-RADS 0 + 4 + 5 (%)	4 (30.8)	9 (69.2)	13 (100)

Kappa 0.784, *p* < 0.0001.

**Table 5:** FFDM+DBT versus FFDM+ABUS.

FFDM+DBT	FFDM+ABUS		Total (%)
	BI-RADS 1 + 2 (%)	BI-RADS 0 + 4 + 5 (%)	
BI-RADS 1 + 2 (%)	28 (100)	0 (0)	28 (100)
BI-RADS 0 + 4 + 5 (%)	9 (40.9)	13 (59.1)	22 (100)

Kappa 0.621, *p* < 0.0001.

**Table 6:** Agreement between the first and the second reader regarding the BI-RADS score given on FFDM+ABUS.

FFDM+ABUS (First Reader)	FFDM+ABUS (Second Reader)		Total (%)
	No of Patients with a Negative BI-RADS Score (%) (BI-RADS 1 and 2)	No of Patients with a Positive BI-RADS Score (%) (BI-RADS 0, 4, and 5)	
No of patients with a negative BI-RADS score (%) (BI-RADS 1 and 2)	36 (94.7)	2 (5.3)	38 (100)
No of patients with a positive BI-RADS score (%) (BI-RADS 0, 4, and 5)	0 (0)	12 (100)	12 (100)

Kappa 0.896, *p* < 0.0001.

**Table 7:** Agreement between the first and the second reader regarding the BI-RADS score given on FFDM+DBT.

FFDM+DBT (First Reader)	FFDM+DBT (Second Reader)		Total (%)
	No of Patients with a Negative BI-RADS Score (%) (BI-RADS 1 and 2)	No of Patients with a Positive BI-RADS Score (%) (BI-RADS 0, 4, and 5)	
No of patients with a negative BI-RADS score (%) (BI-RADS 1 and 2)	23 (88.5)	3 (11.5)	26 (100)
No of patients with a positive BI-RADS score (%) (BI-RADS 0, 4, and 5)	2 (8.3)	22 (91.7)	24 (100)

Kappa 0.8, *p* < 0.0001.

## Data Availability

The data is available only by request.
